# Leucocyte Trafficking via the Lymphatic Vasculature— Mechanisms and Consequences

**DOI:** 10.3389/fimmu.2019.00471

**Published:** 2019-03-14

**Authors:** David G. Jackson

**Affiliations:** MRC Human Immunology Unit, Radcliffe Department of Medicine, MRC Weatherall Institute of Molecular Medicine, University of Oxford, Oxford, United Kingdom

**Keywords:** lymphatic, trafficking, transmigration, dendritic cell, endothelium, chemokine, hyaluronan, LYVE-1

## Abstract

The lymphatics fulfill a vital physiological function as the conduits through which leucocytes traffic between the tissues and draining lymph nodes for the initiation and modulation of immune responses. However, until recently many of the molecular mechanisms controlling such migration have been unclear. As a result of careful research, it is now apparent that the process is regulated at multiple stages from initial leucocyte entry and intraluminal crawling in peripheral tissue lymphatics, through to leucocyte exit in draining lymph nodes where the migrating cells either participate in immune responses or return to the circulation *via* efferent lymph. Furthermore, it is increasingly evident that most if not all leucocyte populations migrate in lymph and that such migration is not only important for immune modulation, but also for the timely repair and resolution of tissue inflammation. In this article, I review the latest research findings in these areas, arising from new insights into the distinctive ultrastructure of lymphatic capillaries and lymph node sinuses. Accordingly, I highlight the emerging importance of the leucocyte glycocalyx and its novel interactions with the endothelial receptor LYVE-1, the intricacies of endothelial chemokine secretion and sequestration that direct leucocyte trafficking and the significance of the process for normal immune function and pathology.

## Introduction

The lymphatics form an extensive network that facilitates the drainage of plasma leaked from the peripheral vasculature and its re-uptake by the venous circulation for maintenance of fluid homeostasis (1, 2). Moreover, they constitute an essential compartment of the immune system, providing conduits for the trafficking of antigen loaded dendritic cells (DCs), memory and regulatory T cells (T_MEM_ and T_REG_) and neutrophils to draining lymph nodes (dLNs) in the process of immune activation, modulation and peripheral tolerance (3–8). In addition, they mediate the clearance of macrophages that remove pathogens and tissue debris during resolution of tissue inflammation and infection, and are exploited by microbial pathogens such as Group A *streptococci, Salmonella, Brucella* and *M. tuberculosis*, and parasitic nematodes that use the lymphatics for host colonization and systemic dissemination (9–13). Understanding the mechanisms by which cells enter and exit lymphatics in tissues and lymph nodes and their detailed choreography will therefore be essential to understanding how such processes help regulate immunity, and how they might be manipulated for therapeutic intervention.

The lymphatic network is quite distinct from the blood vasculature in terms of both structure and physiology. Notably, the lymphatics start as blind-ended capillaries that are freely permeable to fluids, and have discontinuous overlapping junctions pre-adapted to cell transit, unlike the conventional tight junctions that seal most blood vessels (8, 14, 15). In addition, unlike the blood circulation, cell trafficking in most afferent lymphatics involves intravasation rather than extravasation, in keeping with their role in accommodating the passage to dLNs of tissue resident leucocytes and transient immune cell populations recruited from the circulation (3–5, 8, 16–18). Moreover, during entry to lymphatic capillaries, leucocytes are exposed to the very low shear rates associated with interstitial fluid flow, as distinct from the high shear rates experienced during extravasation from blood capillaries (19). Reflecting such different environments, some of the molecular mechanisms for leucocyte entry and trafficking in the lymphatics are quite different to those in blood vessels. Nevertheless, as will be apparent from this present review, some are broadly similar. Just as extravasation of leucocytes from blood is triggered by inflammation and the induced expression of dedicated chemokines and adhesion molecules in the vascular endothelium, so too is the entry of most leucocytes to afferent lymph vessels. Indeed, as discussed later, certain key adhesion molecules are shared by both vasculatures.

**Figure 1:**
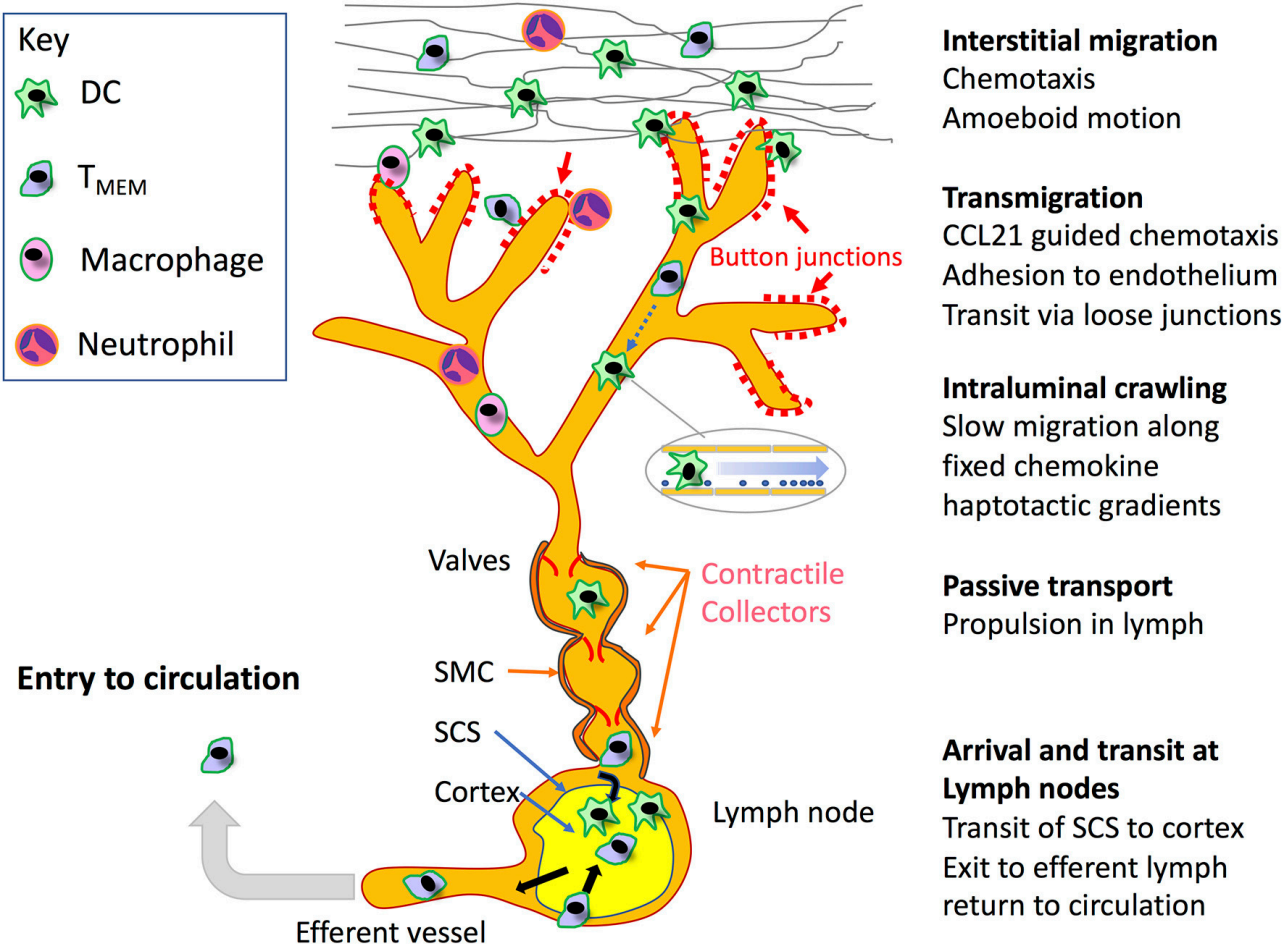
Key steps in leucocyte migration through the lymphatic system. Beginning in the interstitium, motile leucocytes including recirculating (T_RCM_) cells and neutrophils recruited from the blood, in addition to antigen loaded tissue resident DCs and macrophages are mobilized through inflammation to migrate toward initial lymphatic capillaries for access to dLNs by means of chemokine-directed amoeboid movement and in some cases integrin-mediated adhesion to fibronectin. The leucocytes then transit the blind-ended lymphatic capillaries at discrete sites within their discontinuous button-like endothelial junctions through receptor-mediated adhesion and chemokine directed squeezing, or in the case of neutrophils *via* lipoxin mediated chemorepulsion (detailed in Figures 2,4, respectively). Although not depicted, it is also possible that leucocytes can enter downstream vessels through conventional zipper-like junctions, and exit at intermediate stages to the surrounding tissues. On entering the capillary lumen, the transmigrated cells crawl in a semi-directional manner along the endothelial surface, guided by gradients of immobilized CCL21 established under low shear flow, using adhesion to ICAM-1 for traction. On entering downstream valved collectors, the leucocytes are now conveyed by passive transport, propelled by lymph flow generated by smooth muscle contraction. Ultimately, the migrating cells arrive within the SCS of dLNs, where they immediately transmigrate to the underlying cortex to initiate or modify immune responses in the case of DCs and likely neutrophils and macrophages, or continue to the medullary sinuses where they transit to the cortex to either remain there or pass to the efferent lymphatics to re-enter the blood circulation in the case of T_MEMs_. Although not shown in the Figure, naïve T and B cells also recirculate directly from the blood through lymph nodes at specialized high-endothelial venules to enter the cortex and re-exit through efferent lymph.

In the following sections, I describe the latest findings on how leucocytes exploit chemotactic and adhesive mechanisms to enter and migrate within lymphatic vessels, as well as exit the lymphatic sinuses in dLNs to fulfill their various immune functions. I begin with an outline of the major leucocyte populations that migrate in lymph, and the characteristic architecture of lymphatic endothelial junctions. Based largely on knowledge gained from studies on DCs, I go on to provide a detailed account of the key steps in lymphatic trafficking from interstitial migration, lymphatic entry, and intraluminal crawling, to transit within downstream dLNs (Figure 1). In my discourse, I highlight the newly discovered role of the lymphatic endothelial HA receptor LYVE-1 in lymphatic entry and its functional relationship with other more ubiquitous adhesion receptors in endothelial transit, and the possibility of their co-operation in a “lymphatic synapse.” I also describe the co-ordinated triggering of chemokine release by transmigrating DCs, and some of the additional mechanisms employed by neutrophils and certain T cell populations during lymphatic transit. Lastly, I speculate on how knowledge of lymphatic trafficking mechanisms might be exploited in the future to develop new therapies for immune and inflammatory disorders.

## Leucocyte Populations that Traffic *via* Lymph

Classic cannulation studies carried out in domestic animals and applicable also to mice and humans showed the major cell populations migrating in normal afferent lymph are T cells (80–90%), followed by antigen presenting DCs and very small numbers of B cells which together account for most of the remaining 10–15%. Most of the T cells are antigen-experienced CD4^+^ CD45RO^+^ effector memory (T_EM_) cells, recently re-defined as the recirculating memory (T_RCM_) subset (20, 21), which, having entered the extra-lymphoid tissues from blood, engage in immune surveillance for cognate antigens before exiting *via* the afferent lymphatics to dLNs where they modulate recall immune responses (22–25). Notably, lymphocytes of the CD4 subset in afferent lymph outnumber those of the cytotoxic CD8 subset by some 5× fold (26–28), which mostly remain immotile as tissue-resident (T_RM_) cells. Furthermore, more recent cell tracking studies using photoconvertible Kaede mice have revealed that a significant proportion (25%) of the CD4 population are FOXP3^+^ T_REGs_, thus uncovering a previously unrecognized role for the lymphatics in conveying these important immunoregulatory cells. By comparison, only low numbers of naïve T cells are usually present in afferent lymph, and despite the fact these can be shown to enter lymphatic capillaries after adoptive transfer in mice, their normally low frequency in tissue means they rarely do so *in vivo*. The likely functional significance of T_EM_ cell migration in afferent lymph may be to allow the amplification and polarization of immune responses in the dLNs and maintenance of the T cell memory pool, as well as enabling the re-entry of these lymphocytes to the circulation to target pathogen dissemination in further tissue sites (7, 21).

The second most numerous leucocyte population in afferent lymph are DCs, which ferry endocytosed antigens from the tissues, primarily for immune priming in dLNs. Although small numbers of DCs migrate in lymph under steady state conditions to maintain peripheral tolerance to self-antigens (29–33), the majority are mobilized by inflammation, which induces a program of differentiation and the expression of appropriate chemokine receptors for vessel entry (34–36) (see below). DCs, more than any other cell type, have been the subject of studies into the mechanisms of lymphatic trafficking, not least because they are normal tissue residents whose migration can be readily monitored by dye uptake in experimental mice (37). Moreover, in laboratory animals the lymph migrating DCs are almost as numerous as T cells, owing to the fact the pathogen-low environment in which they are bred and maintained generates a smaller pool of circulating memory cells (38).

During inflammation, the numbers of T cells and DCs in afferent lymph increase several fold along with an increase in lymph vessel permeability and the rate of lymph flow (8, 34). Furthermore, the afferent lymph can contain type I and II macrophages which are recruited to inflamed tissues for the clearance of debris and remodeling of the extracellular matrix (ECM) and which utilize the lymphatics for subsequent exit in a process that is becoming increasingly appreciated as critical for resolution and the return to normal homeostasis (39–41). In addition, this lymph contains subsets of neutrophils that are rapidly recruited to the tissues during sepsis and trauma and subsequently exit *via* the inflamed lymphatics to dLNs (42, 43). Although the majority of neutrophils in tissues are short-lived (T_1/2_ 6–12 h) and undergo early apoptosis before removal by macrophage efferocytosis (44–46), the lymph-migrating cells have an extended lifespan (47). Most notably they can transport phagocytosed pathogens such as Leishmania, *H. pylori* and *M. bovis* BCG to dLNs where they can influence the polarity of protective T cell responses through cytokine release and crosstalk with DCs, thus bridging the gap between innate and adaptive immunity (48–51). Indeed, neutrophils can migrate *via* lymph more rapidly than any other leucocyte populations, reportedly arriving in the ipsilateral dLNs some 12–72 h earlier than either DCs or macrophages (52–56).

In contrast to afferent lymph, the leucocyte population present in the efferent lymphatics that exit from the lymph node hilum are mostly naïve T and B cells. Having entered the lymph nodes through high endothelial blood venules in a separate circuit to probe for antigens presented by DCs in the cortex and paracortex, these are ultimately returned to the circulation through the subclavian vein (36). Notably, during the onset of infection or inflammation, the efflux of this recirculating population from the lymph node is halted transiently (3–4 days) so as to prolong their residence time and thus increase the efficiency of immune recognition (57).

## The Distinctive Architecture of Initial Lymphatic Vessels

**Figure 2:**
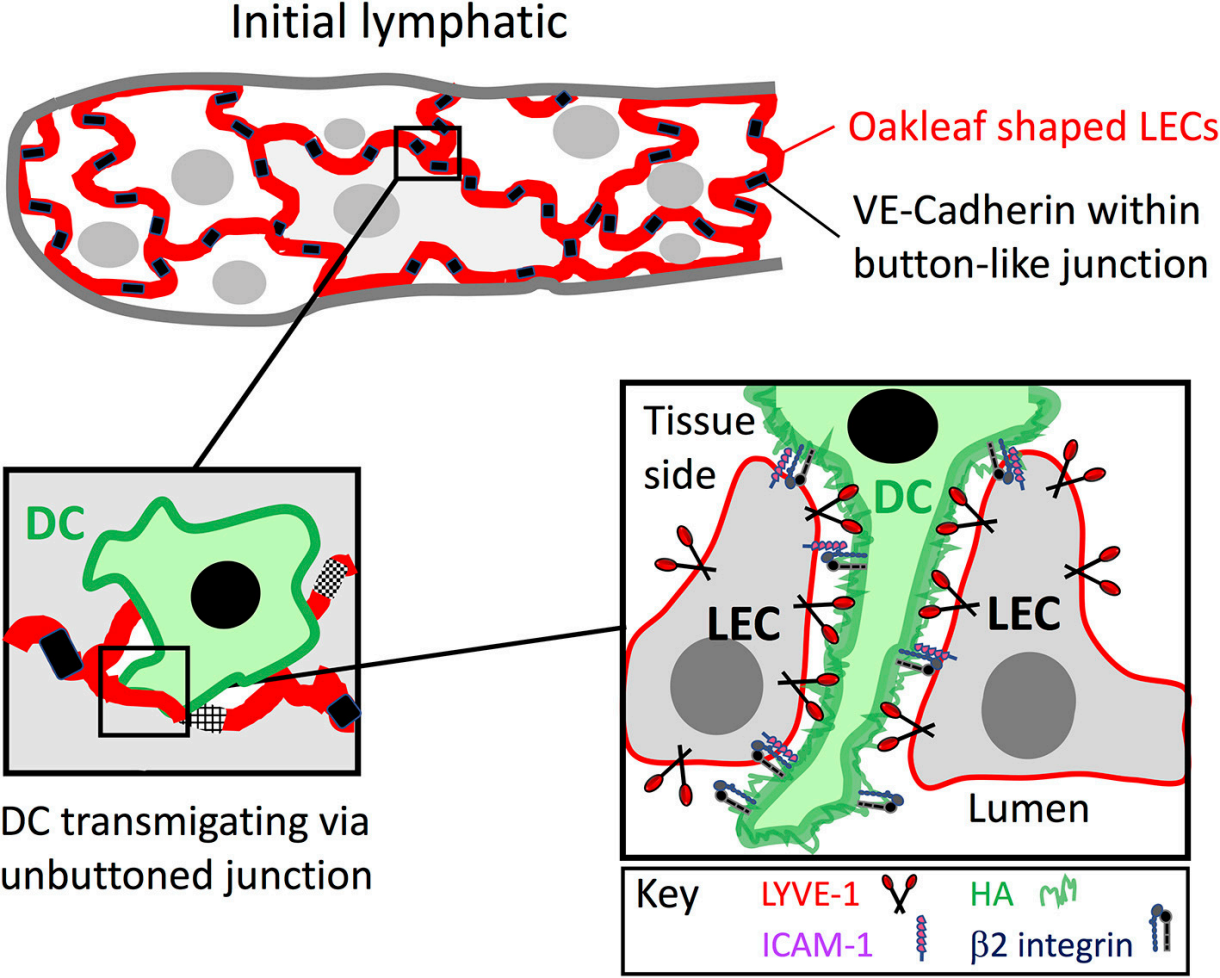
Leucocyte entry to initial lymphatic capillaries through formation of LYVE-1 endothelial transmigratory cups. Entry to the afferent lymphatics proceeds within the discontinuous junctions between the oakleaf shaped lymphatic endothelial cells of initial capillaries that are tightly buttoned at their sides by VE-cadherin and lined at their tips by the HA receptor LYVE-1 (red). As depicted in the Figure, migrating leucocytes such as DCs, macrophages and likely T cells that assemble a surface HA glycocalyx engage with LYVE-1 and transduce signals for VE-cadherin disassembly and junctional unbuttoning that lead to formation of a continuous LYVE-1 lined interface at portals termed transmigratory cups (see text). The weak avidity dependent nature of HA interactions with the LYVE-1 homodimers, combined with the multivalent nature of the long HA polymers are thought to enable low friction migration of the DCs through the endothelial openings. In addition, ICAM-1 and VCAM-1 expressed within the cups bind to integrin ligands on the DC surface activated by local secretion of CCL21 and likely provide the necessary traction for diapedesis. The precise details of the interplay between LYVE-1 and these receptors are not yet known.

As already mentioned, the afferent lymphatics initiate as blind-ended capillaries that branch and merge with larger collecting vessels, emptying their contents into dLNs before exiting as efferent vessels that reconnect either directly, or *via* other intervening nodes to the venous blood (Figure 1). In keeping with their fluid draining function, the blind-ended capillaries have only a rudimentary basement membrane (BM), and lack any investment by actin-containing smooth muscle cells. Moreover, the endothelial cells that make up the first few millimeters of these initial capillaries have a distinctive oakleaf shape that allows them to interdigitate and form loose discontinuous junctions (Figure 2), quite unlike the continuous junctions of endothelia in most blood vessels (14, 58–61). As revealed by electron microscopy (EM) and confocal imaging studies of such capillaries in mice, the alternating membrane flaps making up these structures are pinned at their sides by discrete assemblages some 3 μm wide and spaced 3 μm apart, that contain the adherens-junction protein VE-cadherin and the tight junction proteins Claudin-5, ZO-1 (zonula occludens-1), ESAM (endothelial selective adhesion molecule) and JAM-A (14). In contrast, the flaps remain free at their tips, where they guard openings of ~0.5–1 μm, that are decorated by CD31 and the lymphatic endothelial HA receptor LYVE-1 as detailed below (14). It is through these openings that DCs appear to enter the lymphatic capillaries. Importantly, the process is far from passive, as the dimensions of the DCs are many times greater than the gaps through which they must enter, and hence transit requires pushing and squeezing *via* intimate contact with the endothelium (14, 62–65). While the rationale for such an elaborate arrangement of buttons and flaps is not fully clear, it likely represents a compromise between the conflicting requirements of high vessel permeability for fluid uptake and a more restrictive barrier for regulating leucocyte entry. Indeed, for the experimenter, such architecture poses problems for establishing *in vitro* models to study vessel entry, as primary cultured lymphatic endothelial cell (LEC) monolayers form only partial surrogates of discontinuous junctions (66), and full authentication requires the application of whole animal models or crawl-in assays with tissue explants.

In contrast to the initial capillaries, downstream pre-collector and valved collector vessels lack buttons and instead adopt conventional tight or “zipper” like junctions that allow the formation of a more fully sealed vasculature (14, 15). The collectors are also covered by smooth muscle cells that promote contractile pumping and which convey migrating leucocytes to the dLNs with minimal leakage. Of note, these zippered junctions constitute the default state of capillaries in the developing embryo and early neonate, and button-like junctions emerge only during the later neonatal period, co-incident with the establishment of full immune function (67). Curiously, the nascent lymphatics generated by lymphangiogenesis in chronically inflamed tissues also have conventional zippers, raising the possibility that vessel entry *via* zippers rather than buttons may be permissible in certain contexts (15).

## Migration Through Interstitial Matrix and the Peri-Lymphatic Basement Membrane

In order to access the initial lymphatic capillaries for exit from the tissues and onward trafficking to lymph nodes, leucocytes must first migrate distances of several hundred microns through the surrounding interstitial matrix, a variably dense complex of type I collagen fibrils, fibronectin, hyaluronan (HA), and heparan sulfate proteoglycans (HSPGs) (68). The rate of such interstitial migration is thought to be comparable with that of subsequent migration through afferent lymph capillaries [4.4 vs. 5.7 μm/min in the case of DCs (69) and see below] and the time taken for DCs to navigate to a lymphatic capillary from the point of initial mobilization has been estimated as ~1 h (70). In mice, under conditions of normal homeostasis, the numbers of migrating cells in sites such as skin are small and the populations consist mostly of immature dermal DCs and effector T_MEM_ cells *en route* to lymph nodes during immune surveillance. However, in response to inflammation, as deduced from *in vivo* studies using skin contact sensitizing agents and complete Freund's adjuvant (CFA), this traffic increases significantly. For practical reasons, most studies of leucocyte interstitial migration in the context of lymphatic trafficking have focused on DCs in the mouse dermis and epidermis. Although sessile in the steady state, these cells are subsequently induced to differentiate and crawl toward LVs, in response to inflammatory signals received from cytokines, prostaglandins and leukotrienes released from keratinocytes activated by microbial products and other Toll-like receptor (TLR) ligands (71). The movement of activated DCs is further stimulated by the increase in interstitial fluid flow that is characteristic of tissue inflammation. Nevertheless, the major driving force for leucocyte interstitial migration under resting and inflamed conditions is chemotaxis, directed primarily by the chemokine CCL21 secreted radially from lymphatic endothelium and its G-protein coupled receptor CCR7 on both migrating DCs and T cells (32, 36, 57, 72). Elegant studies by Weber and Sixt using single cell tracking in mouse ear skin revealed DC migration begins in a random fashion until the cells reach a distance of ~90 μm from an initial capillary, at which point their migration becomes directional and persistent (73). This directionality of motion is imposed by fixed, haptotactic gradients of CCL21 secreted constitutively from lymphatic vessel endothelial cells and sequestered by collagen and HSPGs in the surrounding interstitial matrix *via* the positively charged chemokine N-terminal domain (73). Moreover, the gradients fall away with distance from the vessel periphery in such a way as to provide a polarity that can be sensed by DCs in the typical size range of 15–50 μm. Importantly, DC migration under both resting and inflamed conditions is independent of β2 integrin/ICAM-1 adhesion, as demonstrated by seminal studies in mice that compared intact and pan-integrin knockout cells (74). Migration proceeds instead by “amoeboid movement” whereby CCL21 directs the pushing and squeezing of DCs through the 3D collagen matrix, primarily by triggering actin polymerization and actomyosin contraction at the leading and trailing edges *via* the small GTPases Rac1 and Rac2 and nuclear contraction *via* the Rho associated kinase ROCK (69, 74, 75). To date few studies have addressed the mechanisms of interstitial migration employed by leucocyte populations other than DCs. Although in the case of CD4^+^ effector T_MEM_, such migration in CFA inflamed ear dermis was also found to be independent of β2 integrin/ICAM-1 adhesion (76), it was nevertheless reliant on guidance *via* β1and β3 integrin-mediated adhesion to matrix fibronectin, laid down in association with parallel oriented collagen fibers (77). In the looser fibronectin-rich ECM network that is characteristic of inflamed tissues, such adhesion may be a greater requirement for the chemokine driven migration of T cells due to their smaller size and inability to extend dendritic processes.

Lastly, before engaging with the external surface of initial lymphatic capillaries, migrating leucocytes must traverse the surrounding BM, a rudimentary structure comprised mainly of type IV collagen and the non-network forming laminin isoform α4, before they can transmigrate the endothelium to enter the vessel lumen. In contrast to the more close-knit BM of blood vessels (78), the BM of lymphatic capillaries is sparse and highly perforated by gaps of ~1 μm diameter that are completely devoid of any ECM components. Making up 30% of the vessel surface, these gaps offer points through which migrating cells can access the underlying vessel endothelium with the aid of physical expansion (to ~2 μm). Studies of inflamed mouse ear skin indicate that those gaps which overlie sites of vessel entry are marked by discrete deposits of sequestered CCL21 which direct the transit of DCs by chemotaxis/haptotaxis rather than simply chemokinesis. Indeed, migrating DCs have been observed to extend cellular processes toward these CCL21 puncta and apparently make physical contact with them (71). Furthermore, detailed imaging of such events in *ex vivo* crawl-in assays with dermal tissue explants (79) suggested these invadopodia-like protrusions enable DCs to transiently expand the BM portals by physical squeezing without the parallel requirement for proteolytic re-modeling observed during leucocyte transit of blood vascular BMs. On traversing the BM the migrating leucocytes then encounter the lymphatic endothelium next to their interdigitating flaps, which bend inwards to accommodate entry to the vessel lumen (79).

## Transmigration of the Vessel Endothelium and Entry to the Lymphatics

### Integrin-Mediated Adhesion *via* Endothelial Microvilli

Similar to leucocyte transmigration of blood vessel endothelium, transmigration of lymphatic endothelium involves prior adhesive interactions between β2 integrins and their Ig superfamily counter receptors. Of note, early *in vivo* studies using mouse models of skin inflammation and contact hypersensitivity showed that the endothelial leucocyte adhesion receptor ICAM-1 is involved in the migration of epidermal Langerhans cells to skin dLNs (80, 81). Nevertheless, the requirement for adhesion receptors in lymphatic transmigration has been disputed, and one particularly prominent study using pan-integrin (*Int*^−/−^) deficient mice reported that under normal resting conditions, DCs can enter lymphatic vessels and migrate to dLNs independently of all integrins, just as documented for interstitial migration, by means of chemokine-directed amoeboid motion (74). Importantly however, these studies tracked the migration of exogenous DCs that had been adoptively transferred to a non-inflamed dermis and hence reported a mode of trafficking that proceeds only quite rarely in normal tissues (74). Indeed, other studies that tracked endogenous DC trafficking in the uninflamed skin of CD11c YFP^+^/VE-Cadherin Cre/Rosa26 Fl RFP^+^ chimeric mice detected few if any cells entering dermal lymphatic capillaries, and such entry was observed only after adoptive transfer into tissue inflamed by either contact hypersensitization, exposure to adjuvants (CFA) or treatment with bacterial LPS (71). Hence, an integrin-independent mode of lymphatic vessel transmigration likely applies only to the relatively small minority of immature DCs that traffic constitutively in immune surveillance.

In contrast, in inflamed tissues where DCs migrate more extensively *via* lymph, transmigration is indeed dependent on integrin-mediated adhesion mechanisms. As evidenced by studies with cultured primary LECs, and confirmed by transcriptional profiling of lymph vessel endothelium isolated from inflamed mouse skin, exposure to inflammatory cytokines and contact sensitizing agents results in rapid (T_1/2_ ~3 h) upregulation of the key integrin counter-receptors ICAM-1 and VCAM-1 together with various other adhesion molecules associated with leucocyte endothelial transit such as E-selectin, and a range of different chemokines that are attractants for DCs, monocytes, lymphocytes and neutrophils (82, 83). Furthermore, ICAM-1 and VCAM-1 blocking mAbs have been demonstrated to impair adhesion and transmigration of bone marrow DCs (BMDC) across inflamed LEC monolayers *in vitro*, as well as entry and trafficking of endogenous DCs to dLNs *in vivo*, both in murine models of skin hypersensitivity and dermal vaccine-induced T-cell immunity (82, 84). Likewise, in CD45.2 mice, LFA-1 blocking mAbs were shown to impair trafficking of injected CD45.1 DCs to dLNs from TNFα treated footpads (85). Most notably, it has been observed using *in vitro* confocal imaging of murine BMDCs engaged in transit across inflamed lymphatic endothelium, that ICAM-1 and VCAM-1 are concentrated within finger-like projections somewhat analogous to the transmigratory cups described originally in blood vascular endothelium (86–89) (Figure 2 and see below). These extend around the transmigrating cells, facilitating their adhesion *via* activated (mAb 24^+ve^) forms of β2 integrin (LFA-1) present in complementary projections on the DC surface (85). Reportedly, similar ICAM-1 lined membrane protrusions were also observed around transmigrating DCs *in vivo* both in mouse ear and human dermal tissues.

The transmigration of T cells across lymphatic endothelium also involves integrin-mediated adhesion. Accordingly, in studies employing antibody blockade, ICAM-1 and its β2 integrin ligand LFA-1 were shown to be functionally required for T cell adhesion and transmigration of TNFα treated murine LECs *in vitro*, as well as entry to lymphatic vessels and trafficking to dLNs in the inflamed skin of oxazolone and adjuvant treated mice *in vivo* (76). Indeed, in an analogous manner to DCs, ICAM-1 was observed to be distributed in microvillar projections around CD4^+^ T cells and ionomycin/PMA-activated peripheral blood mononuclear cells adhering to a lymphatic endothelium (85). Hence it appears that similar mechanisms are employed by both CD4 T cells and DCs for lymphatic vessel entry through extension of ICAM/VCAM enriched endothelial microvilli (19, 90). Furthermore, as detailed later in this review, the initial stages of neutrophil entry involves adhesion through the β2 integrins LFA-1 and Mac-I and most likely their main lymphatic endothelial counter-receptor ICAM-1, as evidenced from experiments using adoptive transfer of *Int*^−/−^ neutrophils and receptor blocking mAbs (42, 43, 56, 91). Indeed, it was clearly shown that administration of either β2 integrin or ICAM-1 blocking mAbs impaired the entry of GFP*lysM* labeled neutrophils to the initial lymphatic capillaries in mice, causing the cells to logjam at the vessel periphery (56), in a manner reminiscent of DCs given similar blockade in oxazolone-treated mouse skin (17, 82). Such harnessing of integrins, ICAM-1 and VCAM-1 for leucocyte adhesion to the basolateral surface of lymphatic vessel endothelium and subsequent transendothelial migration is in many ways analogous to their involvement in the abluminal crawling of newly extravasated leucocytes on the pericyte surface of blood vessels (92) and stands as an example of how comparable mechanisms can operate in the two vasculatures albeit in reverse orientation.

### Transit *via* Hyaluronan and LYVE-1 Transmigratory Cups

In addition to integrins and their counter-receptors, it has recently been demonstrated that transmigration of DCs requires critical involvement of LYVE-1 (93) within the button-like junctions of initial lymphatic capillaries (14, 15) (Figure 2), and its engagement with the large mucopolysaccharide ligand HA present on the DC surface (19, 90). Closely related to the leucocyte receptor CD44 (94, 95), LYVE-1 contains a conserved lectin-like HA-binding domain, termed the Link module, at its N-terminus (19, 96). Although HA is a ubiquitous component of perivascular ECMs (97), it can also be synthesized by DCs and other leucocytes including macrophages and T cells as a surface glycocalyx (98–100), and it is the selective interaction of LYVE-1 with this latter structure in preference to ambient HA that facilitates DC transmigration (90, 101). Such specificity is possible because of the strict avidity-dependent nature of LYVE-1: HA interactions. Because the receptor binds only a short 8–20 saccharide region of HA with low affinity (K_D_ 125 μM), it therefore relies on homo-dimerization and clustering, as well as a high ligand density to achieve the multiplicity of co-ordinate binding interactions required for tethering of the polymer chains (96, 101, 102), a phenomenon that has been termed superselectivity (103). Intriguingly, *in vitro* confocal imaging studies using primary murine LEC monolayers have shown that LYVE-1 is recruited along with ICAM-1 and VCAM-1 to the transmigratory cups that form upon initial DC contact with endothelium, and furthermore that engagement of LYVE-1 with the HA glycocalyx is actually critical for their formation (90). Consistent with a functional role for the receptor in DC transmigration, LYVE-1 HA blocking mAbs also impaired both the adhesion and transit of DCs across LEC monolayers *in vitro*. Similar features of LYVE-1 transmigratory cups have been observed *in vivo*, during DC transit of lymphatic capillaries in oxazolone sensitized mouse skin. Moreover, interference with LYVE-1 mediated DC interactions in such studies by LYVE-1 gene deletion, antibody blockade or DC HA glycocalyx depletion resulted in the characteristic logjamming of endogenous and adoptively transferred DCs on the basolateral surface of dermal lymphatics, and impaired their capacity to prime antigen specific T cells in dLNs, mirroring the effects seen with β2 integrin blockade (90). Notably, besides DCs, LYVE-1 also mediates adhesion and transmigration of macrophages across lymphatic endothelium through similar mechanisms (102). Indeed, in a murine model of myocardial infarction where damage-inducing M1 macrophages infiltrate the ischaemic myocardium and are subsequently cleared *via* cardiac lymphatics (104, 105), the process is blocked by *Lyve1* deletion, which delays resolution and leads to fibrotic scarring (41). Whether or not T cells also engage LYVE-1 in such structures has yet to be determined. However, in common with DCs and macrophages these also have a capacity for HA biosynthesis, and hence may well assemble a similar HA surface glycocalyx (98). Curiously, another receptor, CLEVER-1, containing an HA-binding “Link” domain related to LYVE-1 has been reported to mediate CD4 and CD8 T cell trafficking through afferent murine skin lymphatics (see Table 1) as well as transmigration across monolayers of lymphatic endothelium *in vitro* (115–117). However, the mode of action of CLEVER-1 is distinctly different to LYVE-1, as the Link module was shown to be non-functional and the site mediating transmigration instead found to reside within a distant EGF repeat region (116, 118).

**Table 1:** Adhesion receptors in lymphatic endothelium involved in regulating leucocyte entry and trafficking.

**Receptor**	**Comment**	**Key references**
ICAM-1	Immunoglobulin superfamily receptor for leucocyte β2 integrin ligands LFA-1 and Mac-1, upregulated in inflammation	See main text
VCAM-1	Immunoglobulin superfamily receptor for leucocyte β1 integrin ligands, upregulated in inflammation	See main text
LYVE-1	Avidity dependent Link superfamily HA receptor binds selectively to migrating leucocyte glycocalyx	See main text
CLEVER-1	Multidomain scavenger receptor in afferent LVs and LN HEVs. Supports adhesion of lymphocytes, monocytes, and granulocytes. mAbs impair migration to dLNs. Ligands yet to be identified	See main text (103–105)
Mannose receptor (CD206)	C-type lectin receptor supports lymphocyte adhesion and migration to dLNs by binding E-selectin and sulphated glycans	(106–110)
ALCAM (CD166)	Mediates DC adhesion to LECs *in vitro* and migration from lung to dLN *in vivo*. Binds CD6, L1CAM, Galectins	(111)
L1CAM (CD171)	Homotypic adhesion molecule expressed in inflamed lymphatics and DCs. Disruption impaired DC endothelial transmigration *in vitro* and trafficking to dLNs *in vivo*	(112)
4-1BB (CD137)	Induced in LEC by TNFα, IL-1, LPS. Ligation potentiates DC transmigration by upregulating ICAM-1, VCAM-1, CCL21	(113)
CD34 (PECAM-1) and CD99	Homophilic adhesion molecules at LEC:LEC junctions and luminal surfaces. Both support DC adhesion/transmigration	(114)

Importantly, the role of LYVE-1 in lymphatic transmigration extends beyond merely supporting cell adhesion. For example, it has been demonstrated that engagement of the receptor can transduce signals for endothelial junctional relaxation, in particular the phosphorylation and detachment of VE-cadherin located within the button-like foci of initial capillaries from which LYVE-1 is selectively excluded (14, 106). Hence, engagement with the DC glycocalyx and un-buttoning of the VE-cadherin lined junctions at vessel entry sites may well promote coalescence of the alternating endothelial flaps and redistribution of LYVE-1 to form a single continuous interface for leucocyte diapedesis (2, 19). In addition, the large contour lengths of HA polymers, which can extend to several microns, likely mask access to the more compact integrins [extracellular domains ~20 nm (107)] on the underlying DC surface, and allow the polysaccharide to make primary contact with the capillary endothelium. It has also been postulated that the low affinity of LYVE-1 HA-binding supports crawling of DCs along the vessel surface toward junctional portals through the inherently low friction of the interaction (19). This role as lubricant is supported by physicochemical studies of LYVE-1 HA binding mechanics at the single molecule level using atomic force microscopy, which indicate the individual interactions are weak and that they rupture collectively under the low forces experienced in interstitial flow [see (19)]. It contrasts markedly with the behavior of CD44, a receptor tuned for leucocyte capture in post-capillary venules, which forms bonds that are stronger and detach sequentially in a Velcro (hook and loop) like fashion in response to the higher forces experienced in blood flow (108, 109). Nevertheless, the transit of cells through lymphatic endothelium must involve traction, and if this is not provided by LYVE-1, then it is likely that DCs use both HA and integrin-based adhesion either on different faces of the cell, or in sequential fashion during diapedesis.

Undoubtedly, many other adhesion molecules located in and around the buttoned junctions of lymphatic endothelium contribute to leucocyte transmigration, and may even specify the entry of discrete leucocyte populations. A number of such receptors including Mannose receptor (110–114), ALCAM (CD166, Activated Leucocyte Cell Adhesion Molecule) (119), L1CAM (CD171) (120), 4-1BB (CD137) (121), CD99, and CD31 (PECAM-1) (122) have already been implicated in the process from various *in vitro* and *in vivo* studies (Table 1). However, the precise functional roles played by each of these receptors, and how they are individually choreographed during lymphatic trafficking have yet to be elucidated.

## Directional Guidance of Leucocyte Transmigration by Chemokines

### The Key Roles of CCL21 and CCR7

In concert with adhesion receptors, critical cues for the guidance of leucocytes during the process of lymphatic vessel entry are provided by chemokines synthesized and secreted in the main by underlying LECs. However, the emerging view is that these may operate as much by inducing the transient arrest of migrating leucocytes at endothelial junctions as by guiding their migration along conventional chemotactic gradients. CCL21 released from the endothelium has been identified as the primary chemokine controlling the entry of DCs to afferent lymph, based initially on the findings from elegant studies in mice showing that CCL21 neutralizing mAbs or CCR7 gene deletion decreased or delayed DC migration from the dermis to dLNs (32, 72, 123, 124). Curiously, mice express two separate genes for CCL21 that encode a lymph node isoform CCL21^ser^ and an afferent vessel isoform CCL21^leu^ (125, 126), and it has logically been assumed (though not formally proven) that the latter (CCL21^leu^) controls the lymphatic entry step (32, 72, 123, 124, 126). Although a naturally occurring genetic deletion of CCL21^ser^ in the *plt/plt* mouse line compromises DC trafficking *via* lymph (125–127), this likely reflects a more distal defect in either entry or retention in downstream dLNs.

In addition to DCs, CCL21 is also the primary chemokine driving the entry of T cells to afferent lymph vessels in the periphery, in particular the antigen-experienced CD4^+^ and CD8^+^ T_EM_ population that exits from the circulation to patrol the inflamed tissues. Approximately 50% of this population in the skin of humans and mice are CCR7^+^, and as confirmed from cannulation studies in sheep, almost all the T cells that migrate in afferent lymph express CCR7 and respond chemotactically to CCL21 (23, 128). Furthermore, parallel studies with CFSE labeled T cells in gene deficient mice have indicated their entry to the afferent lymphatics in both dermis and lung and trafficking to dLNs is almost entirely dependent on expression of CCR7 (20, 23). These migratory CCR7^+^ T cells which can ultimately re-enter the blood *via* the thoracic duct are clearly distinct from the CCR7^−^ T effector (T_EM_) population that remains resident within the tissues as sentinels, and have since been defined phenotypically as a (CCR7^int/+^ CD62L^int^ CD69^−^ CD103^+/−^ E-selectin ligand^+^) recirculating memory (T_RCM_) cell subset, based on their tracking in photoconvertible fluorescent *Kaede* mice (21). Indeed, they also include the important immunosuppressive CD4^+^ T regulatory cell (T_REG_) as well as inflammation-associated Th1 and Th17 cell subsets (129, 130). Curiously however, the importance of CCL21/CCR7 for T cell entry and trafficking is diminished in chronic as compared to acute inflammation (130), and hence it is likely that other inflammation-induced chemokines become involved at later time points (see below).

It has also been reported that CCL21 chemotaxis helps direct the entry of neutrophils to afferent lymphatics. Accordingly, in a study of skin inflammation evoked by topical CFA administration in mice, the lymph migrating neutrophil population was identified exclusively as CCR7^+^ and trafficking to dLNs was decreased almost 4 × fold in CCR7^−/−^ animals (55). Likewise, the entry of neutrophils to the cremaster muscle lymphatics following TNFα treatment, which induces CCL21 release, was reported to be almost completely (>97%) inhibited in CCR7^−/−^ mice (43). Whether CCL21 is the primary chemokine in every context is however open to question, as another study of neutrophil migration in *S. aureus* treated mice found that neutrophil entry to the dermal lymphatics was directed by CXCL12, on the basis of inhibition by the CXCR4 receptor antagonist AMD3100 (91).

### Leucocyte-Induced Chemokine Release

**Figure 3:**
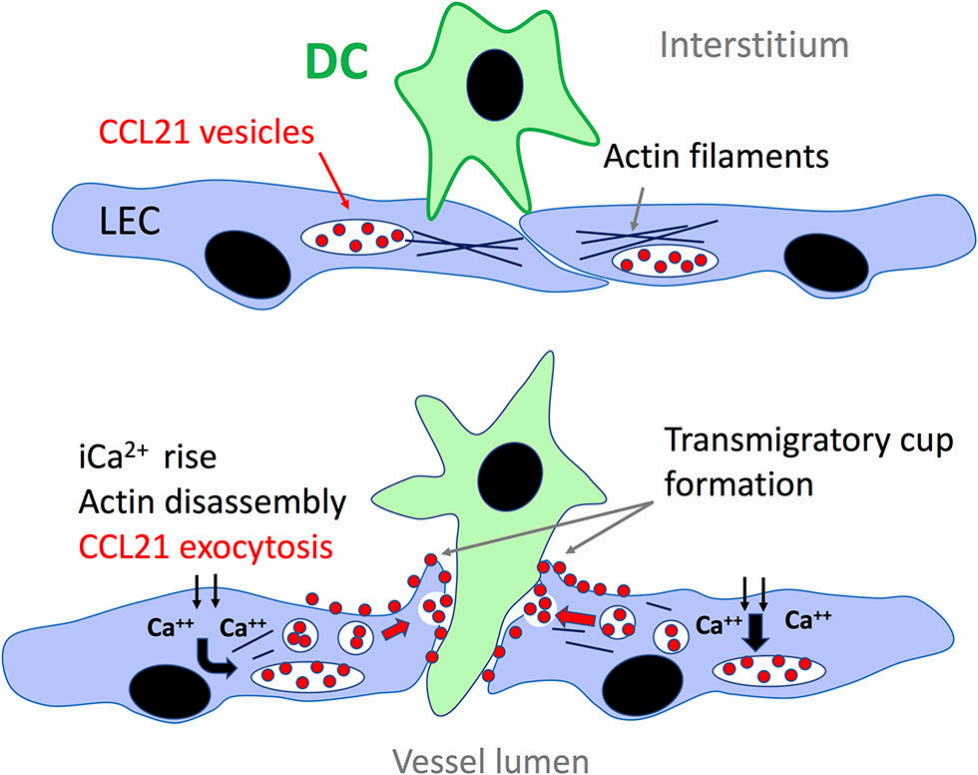
Local secretion of CCL21 from lymphatic endothelium triggered by DC contact. Contacts with the basolateral surface of lymphatic endothelium *via* filopodia extended from the migrating DCs trigger an influx of Ca^2+^ that leads to disassembly of the adjacent cortical actin network and enables translocation of endothelial trans-Golgi vesicles containing pre-stored CCL21 to the plasma membrane along parallel microtubule tracks. Fusion of the vesicles with the plasma membrane, most likely orchestrated by Ca^2+^ binding synaptotagmins, leads to the local release of CCL21 which is retained on the plasma membrane surface close to the endothelium:DC interface, where it directs DC transit through β2 integrin activation and actomyosin-mediated pushing and squeezing, upon the formation of LYVE-1 and ICAM-1/VCAM-1 lined transmigratory cups (see text and Figure 2). The identity of the adhesion molecules involved in making initial contact between DC and lymphatic endothelium are not currently known.

Synthesis of CCL21 is markedly upregulated in murine and human lymphatic endothelium in response to inflammation (35, 66, 131) whereby the chemokine accumulates in intracellular storage vesicles in readiness for secretion, notably at the basolateral surface of the endothelium where leucocytes transmigrate (132). Intriguingly, in a recent seminal study of DCs by Vaahtomeri *et al* it was reported that leucocytes themselves can trigger such secretion from lymphatic vessels through a contact dependent mechanism in which the transmigrating cells extend filopodia toward the endothelium, provoking a Ca^2+^ flux that triggers disassembly of cortical actin and the exocytosis of pre-stored CCL21 from trans Golgi vesicles along linear microtubule tracks, for fusion with the plasma membrane (Figure 3) (65). As visualized using EM, the exocytosed CCL21 is then retained focally in the form of minute puncta at the basolateral surface of the endothelium close to intercellular junctions where they are thought to induce local arrest of DCs through β2 integrin activation. Such confined release may well avoid the desensitization of CCR7 on the migrating cells that might otherwise be evoked by formation of a conventional transendothelial CCL21 gradient (133). Diapedesis—the actual transit process, then proceeds by CCL21-driven re-arrangement of the DC actomyosin cytoskeleton that allows the cell to push and squeeze through the flap-like protrusions between adjacent oakleaf shaped endothelial cells in the initial capillary junctions (65), aided by Semaphorin 3A induced signaling *via* RhoA and ROCK for contraction of the uropod (134). The CCL21 secreted focally in response to DC contact is thus functionally distinct from the CCL21 that is secreted homeostatically for interstitial migration. Interestingly, the DC adhesion-induced CCL21 exocytotic mechanism described by Vaahtomeri et al. does not appear to be initiated by integrins. Indeed, the identities of the receptors on DCs and endothelium responsible have yet to be determined. Moreover, it is likely that the process of DC-induced CCL21 secretion orchestrates the assembly of LYVE-1, ICAM-1, and VCAM-1 enriched endothelial transmigratory cups, as independent studies reported that corresponding structures consistently formed close to CCL21 puncta in LEC monolayers *in vitro*, and their assembly was blocked by CCL21 neutralizing antibody (76, 85, 90, 135). It remains to be determined whether other leucocyte populations such as CD4^+^ T cells can trigger local CCL21 secretion in a similar manner to DCs and whether the coupling of chemokine release and transmigratory cup formation is a general phenomenon for vessel entry.

### Additional Inflammatory Chemokines and Exosome—Mediated Secretion

Besides CCL21, LECs synthesize a variety of other chemokines including CCL1, CCL2, CCL5, CCL20, CXCL12, and CX3CL1 that are chemotactic for T cells, DCs and monocytes expressing the G-protein coupled receptors CCR8, CCR2, CCR5, CCR4, and CX_3_CR, respectively, and CXCL1, CXCL2, CXCL5, and CXCL8 that are chemotactic for neutrophils that express CXCR1 and CXCR2. In common with CCL21 these are all upregulated by exposure of the endothelium to inflammatory cytokines or other inflammatory stimuli (82, 83). In most cases however, the mechanisms underlying their release and extracellular localization in relation to junctional entry sites are not so well-understood. Amongst those chemokines that have been studied in any significant detail, CXCL12 (SDF-1) has been reported to direct the entry of DCs and epidermal Langerhans cells to lymphatic vessels in mice and migration to dLNs as assessed by FITC skin painting (136, 137). More specifically, its receptor CXCR4 was shown to be highly expressed by lymph-migrating MHC class II^+^ DC in skin and co-administration of a synthetic CXCR4 antagonist (4-F-benzoyl-TN14003) impaired their migration to dLNs and capacity to promote T cell proliferation after contact hypersensitization with the hapten DNBS (136). Nevertheless, the authors concluded that the role of CXCL12/CXCR4 axis, although operating in parallel for DC migration was subordinate to that of CCL21 and CCR7. Furthermore, CXCL12 does not appear to control the exit of T cells to afferent lymphatic vessels in inflamed skin, despite the fact they express CXCR4 and exhibit responsiveness to the chemokine *in vitro* (138). Additionally, CCL1, which regulates DC and tumor cell transit across the LN SCS *via* its primary receptor CCR8, has also been implicated in directing transmigration of monocytes and monocyte derived DCs across inflamed peripheral lymphatics (139–141).

More recently, CX3CL1 was shown to promote both *in vitro* transmigration and *in vivo* lymphatic entry of CX3CR^+^ monocyte-derived DCs in the skin of oxazolone-hypersensitised mice in parallel with CCL21 (142). Unique amongst chemokines, CX3CL1 is synthesized as a membrane-anchored molecule that is subsequently cleaved by proteases including the disintegrin and metalloproteases ADAM10 and ADAM17 to generate a conventional soluble chemoattractant, and it is this form that is released basolaterally from cytokine-activated endothelium (142). Moreover, as reported within the last few months (143), CX3CL1 is also secreted from lymphatic vessels in CD9^+^ and CD63^+^ exosomes that form halos around the periphery of lymphatic vessels in inflamed mouse and human tissues. Intriguingly, these exosomes which carry the membrane-anchored form of CX3CL1 on their surface can elicit cellular protrusions in monocyte-derived DCs and promote their transmigration across human dermal LEC (HDLEC) monolayers, in co-operation with CCL21, as well as entry to intact lymphatic vessels in *ex vivo* exposed skin (143). Why lymphatic vessels should employ two such different modes of CX3CL1 release is unclear. However, as exosomes act as vehicles for the release of chemokines other than CX3CL1 it is possible they direct the entry of multiple different inflammatory leucocyte populations.

Interestingly, most of the remaining chemokines such as CCL2, CCL5, CCL20, and the neutrophil chemokines CXCL2, CXCL5, and CXCL8 are preferentially secreted from the luminal rather than the basolateral face of lymphatic endothelium (17, 56, 66, 132, 142), unlike CCL21 and CX3CL1, and hence it is currently unclear how they might regulate leucocyte entry to afferent lymphatic vessels. Indeed, the reduced trafficking of epidermal Langerhans cells and CD8^+^ dermal DCs to dLNs observed in CCR2^−/−^ and to a lesser extent in CCR5^−/−^ mice appeared to result from their accumulation *inside* rather than *outside* dermal lymphatics and hence it is more likely that such chemokines direct intraluminal crawling rather than initial vessel entry (144).

Finally, the steady-state levels of secreted chemokines on and around lymphatic capillaries are regulated by a group of “atypical” chemokine receptors present in lymphatic endothelium that lack signaling capacity and act primarily as chemokine scavengers. This group which includes ACKR1 (Duffy antigen), ACKR2, formerly known as D6, ACKR3 (CXCR7), and ACKR4 (CCRL1) bind and internalize inflammatory CC chemokines and prevent their inappropriate accumulation on the surface of lymphatic capillaries while also helping to establish the polarity of their gradients (145). Accordingly, the action of ACKR2 which scavenges the inflammatory CC chemokines CCL2, CCL3, CCL4, and CCL5 is thought to aid in the preferential entry of mature activated CCR7^+^ DCs *via* CCL21, largely by preventing an accumulation of CCR2/CCR5 macrophages at the vessel surface that might otherwise block their access (146, 147). It has also been posited that ACKR4 helps preserve the responsiveness of DCs to CCL21 during entry to dermal lymphatics in inflamed tissues by scavenging CCL19 released from stromal cells and preventing a build-up of the chemokine that might otherwise de-sensitize CCR7 and lead to DC stasis (148).

### Chemoattractive Guidance for T Cells *via* Sphingosine-1-Phosphate and Lymphotoxins

Besides conventional chemokines, T cells also engage two other chemoattractive pathways for vessel entry, that function co-operatively with CCL21 and may impinge at least partly on the integrin: ICAM/VCAM mediated transmigratory mechanism described above. The first involves the chemotactic lipid sphingosine 1 phosphate (S1P), best known as a regulator of lymphocyte exit from lymph nodes but which appears also to be a negative regulator of CD4^+^ T cell entry to initial lymphatics in inflamed peripheral tissues. Normally present at high concentrations in lymph and low concentrations in lymph nodes, the resulting gradients of S1P direct transit of T cells bearing the G-protein coupled receptor S1PR to efferent lymph (149, 150). However, in a murine alloantigen-induced model of inflammation, increased synthesis of S1P in peripheral tissue or administration of the S1PR1 functional antagonist FTY720 (fingolimod) signals retention and arrest of CD4^+^ T cells, which logjam around the basolateral surface of initial lymphatic capillaries (151). Moreover, as shown using *in vitro* transmigration assays with monolayers of the lymphatic endothelial like cell line SVEC4-10, S1P treatment of T cells blocked their transit and caused their arrest through β2 integrin-mediated adhesion to ICAM-1 and VCAM-1 (151). Whether this S1P/S1PR driven chemotactic mechanism also operates in conjunction with CCL21/CCR7 to positively regulate T cell transmigration and whether ICAM-1 and VCAM-1 are recruited to structures similar to or distinct from DC transmigratory cups during S1P mediated transmigration/arrest remains uncertain.

Secondly, the main immunoregulatory T_REG_ population that migrates between tissues and lymph nodes to maintain peripheral tolerance and immune suppression have been shown to employ lymphotoxin (LT), a lymphokine member of the TNF superfamily, to transit inflamed lymphatic vessels by engaging its signal transducing receptor LTβR in lymphatic endothelium (152, 153). Best known for its role in inducing lymph node neogenesis *via* lymphoid tissue inducer (LTi) cells, the trimeric lymphotoxin molecule is expressed on the surface of T_REG_ as a membrane-bound LTα_1_β_2_ heterotrimer. Moreover, genetic deletion of LTα in murine T_REGs_ or treatment with soluble LTβR Ig fusion protein was shown to disrupt their entry to lymphatic capillaries in mouse skin, while leaving the transit of naïve CD4 and CD8 cells unaffected (153). More specifically, the interaction between LT and LTβR *in vitro* induces the rapid extension of VCAM-1 enriched lamellipodia-like protrusions akin to transmigratory cups in lymphatic endothelium, that appear to facilitate T_REG_ transmigration (153). Curiously however, the VCAM counter-receptor involved in LT-mediated T_REG_ transit has not been identified. While RNA array data suggest that T_REGs_ express much higher levels of LTα than other T cell types (154), it remains unclear whether this mechanism is used by other T or B cell populations for lymphatic transmigration, or indeed whether the LTβR is redistributed to T_REG_ transmigratory structures in lymphatic endothelium together with LYVE-1 and VCAM-1 must await further investigation.

## Transmigration *via* Chemorepulsion—the Unusual Mechanism used by Neutrophils

**Figure 4:**
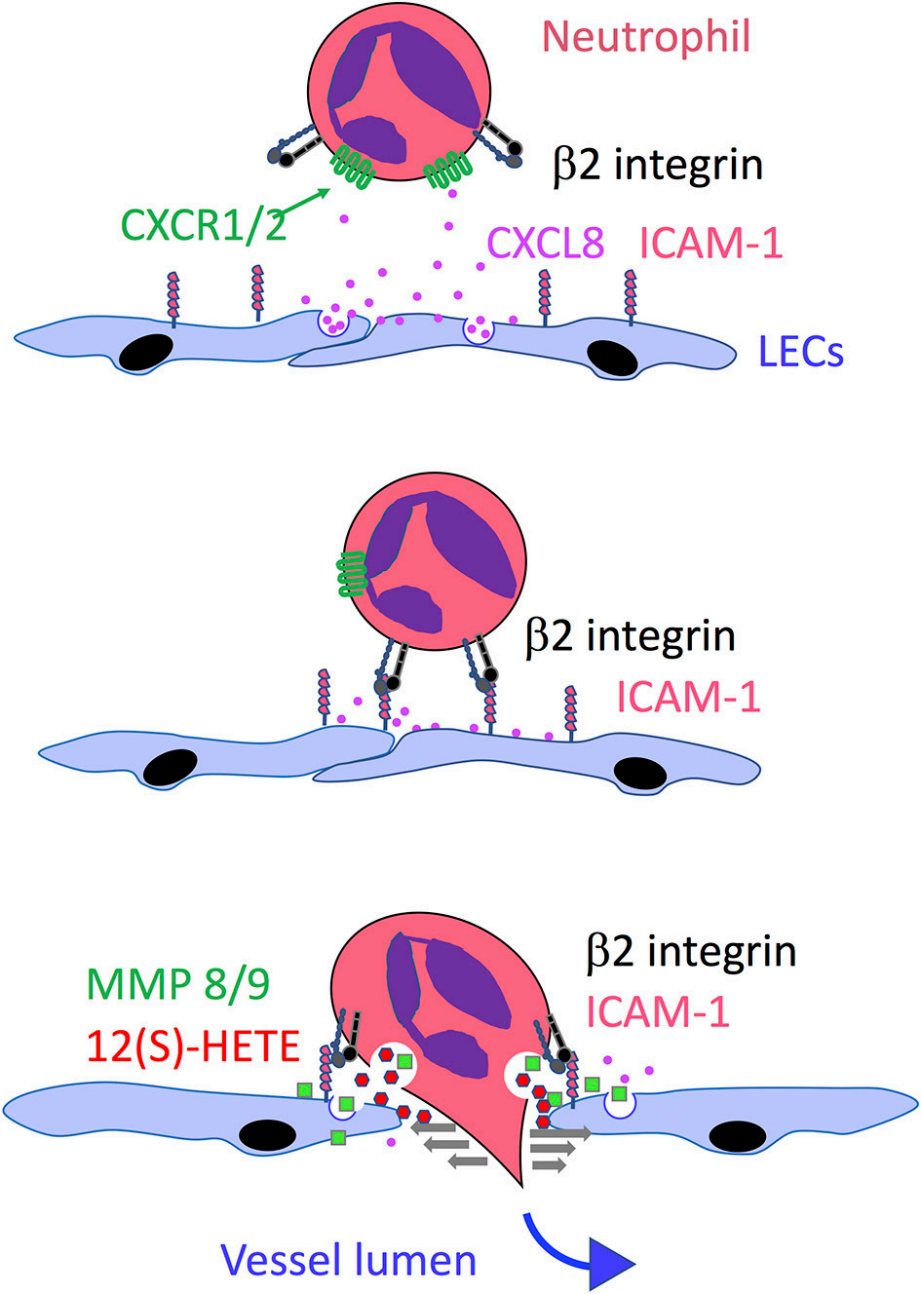
Model of neutrophil transmigration across lymphatic endothelium, Based on a combination of *in vitro* studies with HDLECs and *in vivo* studies in mice, the model depicts the guidance of neutrophils toward the basolateral surface of endothelium following inflammation-induced release of the chemokine CXCL8 (IL-8) which transmits signals through its G protein coupled receptors CXCR1 and CXCR2 to activate the neutrophil β2 integrin LFA-1 and engage its endothelial counter-receptor ICAM-1. This in turn triggers local secretion of the matrix metalloproteinases MMP8 and MMP9 and neutrophil elastase (which also bind to the endothelial surface) and exocytosis of the chemorepellent 12(S)-HETE that together promote transient junctional retraction and neutrophil transmigration.

In comparison to other leucocyte populations, neutrophils deploy a particularly unique and complex mechanism to enter lymphatic capillaries. While studies in mice harboring bacterial infections have shown that entry involves adhesion *via* β2 integrins, like that of DCs and T cells, more detailed *in vitro* studies using inflamed human LEC monolayers and mouse tissue explants revealed that such adhesion is just the first in a co-ordinated series of events that induce release of neutrophil elastase and the matrix metalloproteinases MMP8 and MMP9 and focal secretion of the arachidonate-derived chemorepellant lipid 12-hydroxyeicosatetraenoate (12(S)HETE), that together evoke local endothelial junctional retraction (Figure 4) (56, 155). These studies indicated the openings created by such retraction were transient and resolved spontaneously without any attendant cell death. Moreover, they served as portals for enhanced transit of successive waves of neutrophils which formed loosely attached swarms over the LEC monolayers resembling those previously described in lymph nodes of infected mice. Notably, the rate of *in vitro* neutrophil transmigration was some 10 × fold higher than that of DCs, even in neutrophil pre-exposed LEC monolayers, suggesting they exclusively target such portals for entry without inducing significant endothelial damage (56). The contact dependent nature of this neutrophil-induced activation process is curiously reminiscent of the contact-induced secretion of CCL21 by transmigrating DCs. Indeed, neutrophil adhesion to human LEC monolayers triggered the secretion of several chemokines including CXCL2, CXCL5, and CXCL8 from the basolateral surface that could potentially direct *in vitro* neutrophil transit, although experiments with neutralizing mAbs revealed only CXCL8 (IL-8) fulfilled this role (56). Whether a murine IL-8 ortholog guides neutrophil transmigration similarly in mice along with the recently reported actions of CCL21 and CXCL12 (43, 55, 91, 136, 137) remains to be determined. Importantly, neutrophils as distinct from other lymph migrating leucocyte populations do not synthesize an HA glycocalyx, and hence cannot engage LYVE-1 transmigratory cups for vessel entry. The likely significance of the alternative transmigration mechanism is that it offers a far more rapid mode of lymphatic entry than through the button junctions of initial capillaries, in keeping with the primary function of these cells in the rapid response to tissue injury. It is also noteworthy that an analogous lipoxin-mediated process of endothelial retraction has also been described for lymphatic metastasis of tumors in mice, and certain human cancers (156).

## Intraluminal Crawling

Having transited the lymphatic endothelium, migrating leucocytes enter the vessel lumen and begin their onward journey to the dLNs. In initial capillaries, which are non-contractile and lack smooth muscle investment, the rate of lymph flow has been variably estimated as up to 200–300 μm/min (157, 158), some 2–3 orders of magnitude slower than in blood capillaries and sinusoids, and only marginally exceeding that in the interstitium (69). It is not until these capillaries merge into downstream contractile collectors that the flow rates even approach those of blood vessels. Surprisingly, intravital imaging studies revealed that DCs migrate within initial capillaries (6–8 μm/min) at an even slower rate than lymph itself, and that the majority of newly transmigrated cells are not conveyed by passive flow, but rather crawl along the luminal surface of the lymphatic capillaries until they enter downstream collectors (69–71, 159). Moreover, the crawling leucocytes exhibit semi-random patterns of migration, frequently changing direction before moving downstream (160). Indeed, using time-lapsed microscopy of YFP tagged DCs in the ear tissue of lymphatic reporter mice, it was confirmed that the rate and directionality of intraluminal crawling is almost completely unaffected by changes in lymph flow and even proceeds in its absence (160). Instead, crawling was shown to be driven by chemotaxis/haptotaxis, guided by physical gradients of CCL21 sequestered on the luminal surface of the vessel, as evidenced by confocal and immune EM imaging of mouse dermis, and by the demonstration that its downstream directionality in mouse dermal lymphatics was abrogated by CCL21 blocking mAbs or CCR7 gene deletion in the case of both endogenous and adoptively transferred DCs (160). The spontaneous establishment of such intraluminal gradients was elegantly demonstrated *in vitro* by flow chamber experiments with transfected LECs which affirmed that under levels of shear close to those of afferent lymph (0.015 dynes/cm^2^), fluorescent CCL21 secreted from the luminal surface of the endothelium underwent re-binding downstream to form authentic, directionally oriented gradients for DC migration (160) (see also Figure 1). The macromolecules responsible for sequestering the CCL21 gradient on the endothelium likely include the lymphatic marker podoplanin (161) that reportedly binds the chemokine with high affinity (K_D_ 70 nM) in Biacore analyses (162), and HSPGs, whose digestion or specific knockdown has been shown to disrupt CCL21-dependent DC adhesion to LEC monolayers under flow (163–165). Thus, lymph flow generates the chemotactic gradients which drive intraluminal crawling, rather than conveying cells through physical propulsion. The necessary traction for crawling is provided by β2 integrin-mediated adhesion to ICAM-1 on the luminal surface of the endothelium, whose expression is upregulated in inflammation and whose blockade by mAbs was shown to reduce DC crawling velocity *in vitro* (69). Like DC migration in the interstitium, efficient intraluminal crawling of DCs in inflamed lymphatics also depends on signaling *via* the Rho-associated protein kinase ROCK for dissociation of β2 integrin from ICAM-1 and uropod retraction in a continual process of adhesion and detachment from the endothelium (69).

As regards other leucocyte populations, semi-directional crawling behavior has also been observed for CD4^+^ T cells migrating in the lumen of initial dermal lymphatic capillaries, as evidenced in a recent study that used time-lapsed imaging to track CD2 DsRed fluorescent lymphocytes in *Prox1* GFP mice reporter mice (76). Furthermore, the speed of T cell intraluminal crawling and the degree of motility were both markedly increased in the inflamed dermal lymphatics of skin contact hypersensitised mice, supported again by integrin-mediated adhesive interactions with ICAM-1 on the inner surface of the endothelium (76). Just as described for DCs, these T cells became detached from the luminal endothelium as the initial capillaries merged with downstream collectors where they were passively drawn into lymph flow by vessel pumping. Neutrophils also crawl within the lumen of initial lymphatic capillaries in a mostly downstream direction toward lymphatic collectors, and at broadly comparable velocities (mean 6–13 μm/min) to DCs and T cells. In common with these other leucocyte populations, migration was shown to be directed by haptotactic gradients of CCL21 sequestered on the capillary floor in the direction of lymph flow, with traction provided by β2 integrin mediated ICAM-1 adhesion (91). The faster recruitment of neutrophils to dLNs compared to DCs and T cells *via* afferent lymph may therefore be the product of more rapid mobilization in the tissues, the ability to translocate stored CCR7 from intracellular vesicles rather than relying on *de novo* synthesis (43), and a more efficient mode of endothelial transmigration.

On passing from initial capillaries to the smooth muscle invested pre-collectors and collectors, migrating leucocytes encounter a large increase in lymph flow rate (>1 mm/min) that likely renders intraluminal crawling redundant, and hence it is thought the cells are conveyed toward downstream lymph nodes by passive lymph flow.

## Arrival and Transit at LNs

The major destination for leucocytes migrating through tissue lymphatics is the lymph node, an organ the size of a small bean in most peripheral tissues. With the exception of recirculating T_RCM_ cells which reside transiently before returning to the blood circulation *via* efferent lymphatics, most leucocytes reaching the nodes proceed no further and having fulfilled their immune function, ultimately die there. In either case the cells arrive into the subcapsular sinus (SCS), a labyrinthine compartment continuous with the afferent lymphatics that is situated just beneath the outer capsule of the node. From there, they transit across the SCS endothelium to access the T and B cell-rich cortex, in the case of DCs and neutrophils to prime or re-activate T cell immune responses, and in the case of T_MEM_ and T_REGs_ to influence or downregulate such responses, before they egress and circulate back to the tissues (see Figure 1). While exit from the nodes is known to be directed by the sphingosine 1-phosphate (S1P) receptor S1PR on recirculating cells and S1P in efferent lymph (150, 166, 167), it has often been assumed that cell entry from afferent lymph is a more passive process. However, it is becoming increasingly clear that the SCS endothelium represents a checkpoint for nodal entry that is regulated both by chemokines and adhesion receptors.

In one particularly informative study using mouse eGFP tagged leucocytes microinjected into pre-nodal (popliteal) lymphatics, it was found that DCs and CD4^+^ T cells used separate routes to transit across the SCS (168). Whereas, DCs invariably crossed directly through the floor of the SCS to the cortical zones, T cells instead continued to the adjoining medullary sinuses, previously considered as exit routes from the nodes, before transmigrating to the underlying parenchyma (168). Moreover, DC transmigration across the SCS was shown to be directed by CCR7/CCL21 dependent chemotaxis, whereas CD4^+^ T cells required this chemokine receptor pair only for subsequent haptotactic crawling within the underlying parenchyma (168). Curiously however, when CD4^+^ T cells were co-injected into mice, the former then switched to the SCS route for transmigration, suggesting that DCs in some way remodel the endothelium during transit. More recent work has revealed that the role of CCL21 in directing DC transit can also be aided or augmented by other chemokines. Notably CCL1 released from the floor of the SCS was reported to induce the entry of monocyte-derived DCs to the LN parenchyma *via* its cognate receptor CCR8 (139). More recently, in Th2 immunized mice, the alternative CCR8 ligand CCL8 released from CD169^+^ SCS macrophages was shown to potentiate transit of DCs across the SCS by enhancing CCR7 signal transduction (141). Significantly, polarized expression of the alternative CCL19/CCL21 scavenging receptor CCRL1 (ACKR4) in the ceiling of the SCS was reported to maintain the gradient of CCL21 inside the SCS that drives DC transit, as its deletion led to trapping of DCs within the SCS lumen (169).

Knowledge of the contribution made by adhesion receptors in leucocyte transmigration across the SCS floor is still rather sketchy in comparison with peripheral lymphatics. Curiously, a key role has been identified for the transmembrane protein PLVAP (*aka* Pal-E, MECA 32) that forms diaphragm-like structures in the SCS and certain blood vessels and which primarily regulates the size selective entry of macromolecules to the underlying cortical fibrillar conduit network for presentation by LN HEVs (170). Notably, deletion of PLVAP was found to result not only in the uncontrolled entry of small macromolecules to the cortex, but also the enhanced transmigration of injected splenic T cells. Based on *in vitro* studies with isolated SCS endothelium, it was concluded that PLVAP diaphragms guard entry portals remote from VE-cadherin buttoned junctions, through which T cells transmigrate by extension of their leading edges (170). Whether PLVAP plays a direct or indirect role in the process and also mediates transit of DCs and other leucocytes remains unknown. More recently, transcriptional profiling has identified further candidate receptors that might regulate the differential transit of leucocytes across SCS and medullary sinuses. In particular, the macrophage scavenger receptor MSR1 (CD203) was found to be selectively expressed in SCS, where it was shown to mediate T cell adhesion in *ex vivo* assays with frozen sections. Like PLVAP however, the receptor appears to act as a regulator of SCS transit rather than a gatekeeper, as the process was enhanced not retarded in MSR^−/−^ mice (171). Studies have yet to determine whether transit through SCS or other LN sinuses involves leucocyte adhesion *via* β2 integrins in resting or inflammatory conditions, or interactions between the leucocyte HA glycocalyx and LYVE-1 as in the case of initial lymphatic capillaries. Nevertheless, the observation that transit of DCs is accompanied by marked morphological changes in the floor of the SCS endothelium, including modulation of LYVE-1 and realignment of the SCS-lining CD169^+^ macrophages is indicative that the process is complex and that these and other adhesion receptors may well play contributory roles (36). Finally, it should be stressed that dLN sinuses and the surrounding afferent lymphatic network undergo considerable expansion following antigen challenge or inflammation in peripheral tissues, through a process of lymphangiogenesis driven by VEGF-A released primarily by B cells and macrophages arriving through afferent lymph (172–174). This is accompanied by a transient increase in LN size, cellularity and lymph flow that induces DC mobilization and migration, and markedly enhances DC transit into the deep underlying LN cortex and paracortex for lymphocyte activation (174–176). It is highly likely that such changes also enhance the entry of other migrant leucocyte populations to dLNs and it is hoped that future research in this area will yield much needed insights into the underlying mechanisms.

## Summary and Conclusions

Over the past decade, research using new techniques and animal models for tracking and imaging cell migration, combined with the efforts of a wide interdisciplinary community of interested scientists, has led to huge advances in our understanding of leucocyte trafficking in the lymphatic system and its immune significance. As this pace of advance seems set to continue in the immediate future, we can anticipate that the resulting mechanistic insights will translate into new targets and therapies for immune disorders and even the treatment of lymph metastasising cancers.

As outlined in this text, we now have detailed insight into the first key step in such trafficking, the entry of cells to the lymphatic vessels. In the case of DCs, this has revealed an intricate and closely co-ordinated mechanism in which physical contact of migrating leucocytes with lymphatic endothelium triggers the local exocytosis of CCL21 and formation of LYVE-1^+^ transmigratory cups which envelop the migrating cells and promote their transit into the vessel lumen. Moreover, parallel observations that transmigrating T cells and macrophages elicit the formation of similar endothelial protrusions containing ICAM-1, VCAM-1 and/or LYVE-1, and reliance on an HA glycocalyx or β2 integrin adhesion, raise the possibility that lymph-migrating leucocytes exploit a common mechanism for vessel entry (76, 85, 101). Indeed, this could be considered as a form of “lymphatic synapse,” through which appropriate input from other chemokine receptors or signaling components such as lymphotoxin/LTβR and S1P/S1PR1 might direct selective entry of T_REGs_ or other lymph migrating cell populations including Natural Killer (NK) cells and innate lymphoid cells (ILCs). However, the notion of a synapse may not apply to neutrophils which use a unique mechanism of integrin-dependent proteolysis and lipoxin-mediated endothelial retraction to “invade” lymphatic vessels. This unusually specialized process may have evolved to enable these professional phagocytes to exit almost instantaneously from sites of infection and reach the dLNs well-ahead of slower migrating DCs. In comparison, we know little about how the docking and adhesion of transmigrating leucocytes is choreographed and how the many “accessory” adhesion molecules including the Mannose receptor, ALCAM, CLEVER-1, CD31, CD99, and others (Table 1) integrate with key cup-forming components such as ICAM-1, VCAM-1, and LYVE-1. Though most leucocytes that employ such cups have been observed to enter the afferent lymphatics at button junctions in initial vessels, it seems unlikely that these are the only junctional types to allow entry. During inflammation-induced lymphangiogenesis for example, newly sprouting vessels assemble zippered rather than buttoned junctions, and in chronic inflammation, when leucocyte traffic *via* lymph is markedly increased, zippers replace buttons. It will be interesting to determine whether leucocytes have the ability to enter through zipper junctions and whether the process involves different molecular mechanisms to buttons and transit *via* a transcellular or paracellular route. A further priority will be to ascertain whether these mechanisms of lymphatic entry are universally applicable or vary between tissue beds such as the intestines, brain and central nervous system (177), given that most of our current insight has been gained from studies on mouse dermis, due to its greater accessibility.

Recent research has also provided surprising insight into how leucocytes, having entered the initial lymphatics, migrate within the vessel lumen toward downstream lymph nodes. Rather than being conveyed by passive lymph flow, it is now apparent that DCs, CD4^+^ T cells and neutrophils actively crawl along the internal surface of initial vessels using guidance from CCL21 and transient integrin-mediated adhesion. Yet why such mechanisms should have evolved to deliberately slow the downstream progress of antigen presenting and immune effector cells is unclear. Might the intimate contact with endothelium imposed by intraluminal crawling enable for example *en route* uptake of foreign antigens or maturation signals by DCs, or MHC-mediated antigen presentation to recirculating T cells or T_REGs_ for immune tolerance? Could it provide a platform for interactions between different leucocyte populations themselves? Or might intraluminal crawling provide DCs or T cells with the option to exit and re-enter lymphatic vessels prior to reaching dLNs in order to sample or respond to antigen in the surrounding tissues? Though not yet reported in initial lymphatics vessels, there is evidence that migrating leucocytes exit collectors in adipose tissue during bacterial infection, and that DCs which interdigitate the vessel endothelium to regulate vessel permeability and sample the surrounding tissue can subsequently detach and migrate to dLNs (178).

With the many insights into lymphatic trafficking and its immune consequences that have been gained from basic research, there is increasing scope for clinical translation and the development of new immune based therapies based on migration blockade. Judicious targeting of integrins, ICAM-1, chemokine receptors, S1P and particularly lymph-specific adhesion molecules such as LYVE-1 by appropriate blocking mAbs may be envisaged as therapeutic strategies for transplant rejection, to prevent DC migration from engrafted tissues and consequently activation of alloimmune responses in host dLNs. This is particularly applicable in the case of corneal allografts, where such therapies could be applied locally, thus avoiding off-target effects associated with systemic antibody administration. In the case of T_REGs_, the inclusion of lymphotoxin blockade to impair their migration to lymph nodes for immune suppression might also provide an adjunct to checkpoint inhibitors for tumor immunotherapy. As a corollary, our understanding of the main factors regulating DC migration from inflamed tissues could be exploited to optimize vaccine delivery. For many years the notion of using DCs as adjuvants for adoptive cancer immunotherapy has fuelled efforts to enhance their maturation *ex vivo* using TLR ligands and inflammatory cytokines and to optimize antigen loading and presentation. Combining these approaches with pre-conditioning of vaccination sites to boost lymphatic vessel density, the efficiency of DCs for vessel entry and nodal transport should lead to much greater clinical efficacy. Likewise, boosting the exit of macrophages *via* lymphatics could aid in the resolution of inflammation and tissue recovery in conditions such as myocardial infarction where their delayed removal results in a failure of cardiomyocyte replenishment, and an increase in tissue scarring and fibrosis (41, 179–181).

## Author Contributions

The author confirms being the sole contributor of this work and has approved it for publication.

### Conflict of Interest Statement

The author declares that the research was conducted in the absence of any commercial or financial relationships that could be construed as a potential conflict of interest.
